# Early experience with universal preprocedural testing for SARS-CoV-2 in a relatively low-prevalence area

**DOI:** 10.1017/ice.2020.398

**Published:** 2020-08-03

**Authors:** Sarah S. Lewis, Becky A. Smith, Ibukunoluwa C. Akinboyo, Jessica Seidelman, Cameron Wolfe, Allan B. Kirk, Gavin Martin, Thomas Denny, Bruce Lobaugh, Catherine Rehder, Diana Cardona, Mark J. Lee, Christopher R. Polage, Michael B. Datto

**Affiliations:** 1 Division of Infectious Diseases, Department of Medicine, Duke University, Durham, North Carolina; 2 Division of Pediatric Infectious Diseases, Department of Pediatrics, Duke University, Durham, North Carolina; 3 Department of Surgery, Duke University, Durham, North Carolina; 4 Department of Anesthesia, Duke University, Durham, North Carolina; 5 Duke Human Vaccine Institute, Duke University, Durham, North Carolina; 6 DUHS Clinical Laboratories, Department of Pathology, Duke University, Durham, North Carolina

## Abstract

We implemented universal severe acute respiratory syndrome coronavirus 2 (SARS-CoV-2) testing of patients undergoing surgical procedures as a means to conserve personal protective equipment (PPE). The rate of asymptomatic coronavirus disease 2019 (COVID-19) was <0.5%, which suggests that early local public health interventions were successful. Although our protocol was resource intensive, it prevented exposures to healthcare team members.

Asymptomatic or minimally symptomatic infection with severe acute respiratory syndrome coronavirus 2 (SARS-CoV-2) is common, and transmission from asymptomatic and presymptomatic individuals occurs in community as well as congregate living settings.^1,2^ These features of SARS-CoV-2 distinguish it from other respiratory viruses and pose substantial challenges to healthcare infection prevention programs. Notably, symptom-based screening protocols, traditionally used to risk-stratify patients as likely or unlikely to have transmissible respiratory viral infections, do not reliably rule out the possibility of coronavirus disease 2019 (COVID-19). Further complicating matters, SARS-CoV-2 has been found in high concentrations in the upper airways of asymptomatic individuals, at viral copy numbers similar to patients with symptomatic infections.^1^ Thus, healthcare workers who perform procedures that are high-risk for generating aerosols are at risk of exposure from patients with asymptomatic COVID-19.

Recognizing this potential risk, a number of professional societies have recommended use of personal protective equipment (PPE) including respirators and/or testing asymptomatic patients for COVID-19 prior to undergoing high-risk procedures.^3–5^ As the COVID-19 pandemic progressed in our local community and our supplies of PPE, especially N95 respirators, were projected to reach critically low levels, we implemented preprocedure SARS-CoV-2 testing as a means to risk-stratify patients prior to undergoing urgent or time-sensitive procedures to conserve PPE resources and to provide a level of comfort to healthcare workers during a rapidly evolving and uncertain time. Herein, we describe our implementation and early findings of preoperative and preprocedure SARS-CoV-2 testing at Duke Health.

## Methods

Duke Health consists of a tertiary-care academic hospital, 2 community hospitals, and >180 primary care and specialty clinic practices in 10 counties in North Carolina, providing ~70,000 inpatient hospitalizations and 2.4 million outpatient visits annually.

### SARS-CoV-2 testing

The Duke Clinical Microbiology Laboratory validated and implemented 5 in-house SARS-CoV-2 assays as part of our COVID-19 response: (1) the Centers for Disease Control and Prevention’s 2019—nCoV real-time RT-PCR diagnostic panel on March 19, 2020; (2) the Diasorin Simplexa COVID-19 direct assay on March 24, 2020; (3) the Cepheid Xpert Xpress SARS-CoV-2 test on March 31, 2020; (4) the Abbott Molecular Abbott real-time SARS-CoV02 assay on April 2, 2020; and finally (5) the Abbott Diagnostics Scarborough ID NOW COVID-19 on April 9, 2020. During the validation process, the accuracy, limit of detection, and repeatability (precision) of each assay were determined to be consistent with the manufacturers’ assertions in their Emergency Use Authorization (EUA) applications to the US Food and Drug Administration (FDA). Across all platforms, we achieved a daily sustainable capacity to test ~800 tests per day.

During the early phase of the COVID-19 pandemic, in-house tests were prioritized for symptomatic patients who required admission and symptomatic healthcare workers providing direct patient care. On March 31, 2020, we expanded in-house testing to include asymptomatic patients undergoing urgent or time-sensitive procedures. Initially, only patients undergoing nonemergent surgical procedures were included; once point-of-care testing became available, all surgical patients were included in the testing protocol.

### Preoperative and preprocedural testing and recommendations for PPE

The following procedure categories were included in our protocol: all operating room procedures, bronchoscopy, transesophageal echocardiography, electrical cardioversion, electrophysiology procedures requiring general anesthesia, electroconvulsive therapy, upper and lower endoscopy, fluoroscopically guided enteric tube placements, and interventional radiology procedures requiring anesthesia. Patients who were undergoing outpatient procedures were scheduled to have preprocedural nasopharyngeal swabs (NP) collected at one of our outpatient drive-through test sites within 72 hours of the scheduled procedure. Inpatients had NP swabs collected within 2 days of the scheduled procedure. Patients who had a negative SARS-CoV-2 NP swab test result within 7 days of the scheduled procedure and remained asymptomatic were not required to undergo repeat preprocedural testing. In the event that emergent or urgent procedures could not be safely delayed until results of SARS-CoV-2 testing returned, the procedures were completed with proceduralists wearing an N95 respirator or PAPR.

### Data analysis

For the purposes of this analysis, we included only the first SARS-CoV-2 test result for an individual patient and excluded tests ordered on inpatients with stays >14 days. Patients who had a ‘SARS-CoV-2 preoperative screen’ test completed or who had another SARS-CoV-2 test performed within 7 days prior to a perioperative event were included in the preprocedural test group. We used descriptive statistics to describe patients with SARS-CoV-2 tests during the early phase of the COVID-19 pandemic in our region.

## Results

From March 19, 2020, through April 20, 2020, 486 of 8203 SARS-CoV-2 tests (5.6%) were positive. Percent positive rates remained stable from week to week of the outbreak but varied by patient care location; it was highest among patients presenting to the emergency department (151 of 1,898, 7.4%) compared to outpatient locations (321 of 5,533, 5.5%) and inpatient locations (14 of 772, 1.8%).

From March 31, 2020, through April 20, 2020, 6 of 1,580 patients who underwent preprocedural testing (0.4%) had positive results. Notably, no patients were recognized to have symptoms of COVID-19 at the time testing was ordered. However, after the tests returned positive, further questioning revealed that 1 patient had had unexplained fatigue and mild nasal congestion in the week prior to testing, 1 patient had had a flu-like illness in the previous 2 weeks that resolved prior to testing, and a third patient had had symptoms of dyspnea and fatigue prior to testing and developed fever after the test returned positive. Notably, all patients were living independently in the community prior to the outpatient test or acute hospitalization.

## Discussion

We report our early experience with universal SARS-CoV-2 testing of patients undergoing surgeries and high-risk aerosol-generating procedures within a large healthcare system. Our approach was adopted as a strategy to enhance the safety of healthcare providers in the face of critically low PPE supplies while maintaining necessary surgeries and procedures during the COVID-19 pandemic. Overall, this was a laboratory resource-intensive strategy, and 263 tests needed to be performed to identify 1 SARS-CoV-2 infected patient. Although it is unknown if all patients with positive preprocedural tests would have been infectious at the time of their upcoming procedure, preprocedural testing may have prevented an average of 2 high-risk exposures to healthcare worker teams per week of implementation. Due to differences in local community prevalence, on-site testing capacity, and PPE reserves, our findings may not be generalizable to all settings.

Notably, the prevalence of SARS-CoV-2 infection among asymptomatic patients presenting for procedures was lower than reported rates of asymptomatic infection among other patient populations in areas of higher community prevalence.^6^ The low rate of positivity we observed among asymptomatic patients presenting for surgical procedures presumably reflects an overall low incidence of infection in our community and suggests that early public health policy measures to minimize community transmission may have been effective. North Carolina implemented a number of public health measures: restricting mass gatherings and closing educational facilities on March 14, 2020; closing bars and restaurants on March 17, 2020; and closing other nonessential services and issuing a stay-at-home order on March 30, 2020. The weekly percentage of tests returning positive remained <6% during the 5 weeks of the observation period. In fact, most patients who required admission to our facilities with COVID-19 could be linked to known exposures or outbreaks in local prisons and skilled nursing facilities.

Our experience highlights the fact that screening for symptoms or epidemiologic exposure history is imperfect and is not adequate to rule out the possibility of SARS-CoV-2 infection; none of the 6 patients with SARS-CoV-2 infection reported known exposures to other individuals with COVID-19, lived in congregate living facilities, or were recognized to have typical clinical features of COVID-19 at the time testing was performed.^7^ At this time, we plan to continue preprocedural SARS-CoV-2 testing to determine the need for respiratory protection for healthcare providers during aerosol-generating procedures and to inform infection prevention practices in the perioperative period.

However, using preprocedural testing to determine the need for respiratory protection is not without limitation. Because the duration of PCR-positivity outlasts the duration infectivity in some patients, not all positive preoperative tests represent a true transmission risk to the procedural team members. Additionally, all tests can have false negatives, including laboratory and point-of-care tests, and testing as a substitute for enhanced PPE is only appropriate in settings where disease prevalence is low and the negative predictive value of testing is high.^8^ Thus, we plan to re-evaluate the utility of this approach as local prevalence of SARS-CoV-2 and our PPE availability change.

In summary, we report the implementation of universal preprocedural SARS-CoV-2 testing of patients undergoing necessary surgeries and procedures during the early phase of the COVID-19 pandemic. The success of our universal preprocedural SARS-CoV-2 testing program depended on the availability and rapid turnaround time of in-house SARS-CoV-2 testing. Universal screening of asymptomatic patients prior to high-risk aerosol-generating procedures allowed us to conserve critical PPE supplies when our community prevalence of SARS-CoV-2 was relatively low, and our experience may be useful to others who are facing similar PPE supply shortages and aiming to balance staff safety with patient access to care in the midst of the COVID-19 pandemic.
